# Hepatoprotective and Antioxidant Potential of Organic and Conventional Grape Juices in Rats Fed a High-Fat Diet

**DOI:** 10.3390/antiox3020323

**Published:** 2014-04-30

**Authors:** Iselde Buchner, Niara Medeiros, Denise dos Santos Lacerda, Carlos Augusto B. M. Normann, Tanise Gemelli, Paula Rigon, Clovis Milton Duval Wannmacher, João Antônio Pegas Henriques, Caroline Dani, Cláudia Funchal

**Affiliations:** 1 Centro Universitário Metodista do IPA, 90420-060 Porto Alegre, Brazil; E-Mails: iseldeb@ig.com.br (I.B.); niarasm@yahoo.com.br (N.M.); denidsl@yahoo.com.br (D.S.L.); cabmnormann@hotmail.com (C.A.B.M.N); caroline.dani@metodistadosul.edu.br (C.D); 2 Departamento de Bioquímica, Universidade Federal do Rio Grande do Sul, 90035-003 Porto Alegre, Brazil; E-Mails: tantangemelli@gmail.com (T.G.); clovisdw@ufrgs.br (C.M.D.W.); 3 Departamento de Ciências Morfológicas, Universidade Federal do Rio Grande do Sul, 90040-060 Porto Alegre, Brazil; E-Mail: paula.rigon@ufrgs.br; 4 Departamento de Biofísica, Centro de Biotecnologia, Universidade Federal do Rio Grande do Sul, 91501-970 Porto Alegre, Brazil; E-Mail: pegas@cbiot.ufrgs.br; 5 Instituto de Biotecnologia, Universidade de Caxias do Sul, Caxias do Sul, 95070-560 Porto Alegre, Brazil

**Keywords:** liver, antioxidants, grapes, high-fat diet, free radicals

## Abstract

The objective of this study was to investigate the antioxidant and hepatoprotective effect of the chronic use of conventional (CGJ) or organic (OGJ) grape juice from the Bordeaux variety grape on oxidative stress and cytoarchitecture in the liver of rats supplemented with a high-fat diet (HFD) for three months. The results demonstrated that HFD induced an increase in thiobarbituric acid-reactive substances (TBARS), catalase (CAT) activity and 2′,7′-dihydrodichlorofluorescein (DCFH) oxidation and a decrease in sulfhydryl content and superoxide dismutase (SOD) and glutathione peroxidase (GPx) activities. HFD also induced hepatocellular degeneration and steatosis. These alterations were prevented by CGJ and OGJ, where OGJ was more effective. Therefore, it was concluded that HFD induced oxidative stress and liver damage and that the chronic use of grape juice was able to prevent these alterations.

## 1. Introduction

Nowadays, more than 30 million people die each year due to chronic diseases, such as cardiovascular diseases, diabetes, metabolic syndrome and cancer [1]. Among the risk factors for the development of these diseases are hypertension, excess alcohol intake, hereditary factors, smoking, sedentary lifestyle, stress and obesity [2,3,4]. In this context, visceral fat accumulation plays an important role in the associated deleterious effects of excess body fat, including dyslipidemia and hepatic steatosis [5,6]. Although the complex relationship between visceral fat accumulation and hepatic steatosis is not completely understood, it is well described that an imbalance in lipid metabolism in the liver is associated with hepatic steatosis [7,8]

On the other hand, several lines of evidence have shown that a diet rich in fruits and vegetables is linked to a lower incidence of many chronic diseases [9,10,11,12]. Moreover, some studies have also shown an inverse relationship between the consumption of polyphenol-rich foods and beverages and the risk of oxidative stress-induced diseases [9,10,13,14].

Grapes are a rich dietary source of polyphenolic compounds, which have beneficial effects on human health, including antioxidant, cardioprotective and hepatoprotective effects [15,16]. It has already been reported that grape juice compounds can prevent platelet aggregation, Low density lipoprotein (LDL) oxidation, oxidative damage to DNA, coronary diseases, atherosclerosis and brain oxidative damage [17,18,19,20]. Currently, in Brazil, it is possible to find two types of grape juices, the organic (free of pesticides and genetic engineering) and the conventional (traditionally cultivated with pesticide use and/or genetic engineering) [20,21].

Considering that in the last few years, some research has focused on the potential biological effects of polyphenols on the prevention of some chronic diseases [10,14,22,23] and that supplementation with dietary polyphenols reduces body weight gain and prevents hepatic steatosis in rodents fed a hypercaloric diet [24,25,26], the aim of this study was to investigate the antioxidant and hepatoprotective effect of the chronic consumption of purple grape juice (organic and conventional) from the Bordeaux variety on some parameters of oxidative stress in the liver of rats supplemented with a high-fat diet (HFD).

## 2. Experimental Section

### 2.1. Chemicals

All chemical reagents were purchased from Sigma (St. Louis, MO, USA), except for thiobarbituric acid, which was from Merck (Darmstadt, Germany).

### 2.2. Diets

The animals received a standard diet of Nuvilab^®^ (Colombo, Paraná, Brazil) or HFD from Pragsoluções Biosciences (Jau, São Paulo, Brazil). In terms of percentage of energy, the standard diet contained 25.5% protein, 11.7% fat and 62.8% carbohydrate, while the HFD composition was 21% protein, 59% fat and 20% carbohydrate. The total energy of the standard diet and HFD was, respectively, 3440 and 5121 kcal/kg.

### 2.3. Grape Juices

The purple grape juice samples used in this study were from *Vitis labrusca* grapes, Bordeaux variety. Organic grape juice (OGJ) was produced with grapes cultivated without pesticides, obtained from Cooperativa Aecia (Antonio Prado, Rio Grande do Sul, Brazil) and it was certified by Rede de Agroecologia ECOVIDA. Conventional grape juice (CGJ), produced with grapes cultivated using traditional methods, was obtained from Vínicola Perini (Farroupilha, Rio Grande do Sul, Brazil). Validity periods were observed, and the same brands were used for the entire study. Grape juices were manufactured in 2010. The juices were produced by heat extraction (approximately 50 °C), with subsequent pressing in order to separate the pulp, and then submitted to pasteurization (at 85 °C). All juices were manufactured by heat extraction, immediately afterwards followed by bottling at 80 °C. The composition of both grape juices is described as follows. CGJ: total acidity, 0.72 ± 0.014 (g% tartaric acid); volatile acidity, 0.020 ± 0.0001 (g% tartaric acid); total sugars, 12.60 ± 0.141 (g/100 g); moisture, 153.37 ± 0.212 (g/L); ash, 3.30 ± 0.282 (g/L); total phenolic content, 72.30 ± 0.141 (mg catechin/mL); resveratrol, 0.210 ± 0.028 (ppm resveratrol). OGJ: total acidity, 1.01 ± 0.007 (g% tartaric acid); volatile acidity, 0.030 ± 0.0001 (g% tartaric acid); total sugars, 11.67 ± 0.106 (g/100 g); moisture, 143.30 ± 0.141 (g/L); ash, 2.46 ± 0.615 (g/L); total phenolic content, 101.19 ± 0.021 (mg catechin/mL); resveratrol, 0.850 ± 0.014 (ppm resveratrol).

### 2.4. Animals

Forty male Wistar rats, 21 days old, were obtained from our own breeding colony. They were maintained at 22 ± 2 °C, on a 12 h light/12 h dark cycle, with free access to food and drink. The guidelines in “Principles of laboratory animal care”, National Institutes of Health publication No. 80-23, revised 1996, were followed in all our experiments, and the research protocol was approved by the Ethical Committee for Animal Experimentation of the Centro Universitário Metodista (008/2011). All efforts were made to minimize animal suffering and to use only the minimal number of animals necessary to obtain reliable scientific data.

### 2.5. Treatment

The animals were randomly divided into four groups: standard diet + water (Group 1), HFD + water (Group 2), HFD + CGJ (Group 3) and HFD + OGJ (Group 4). The animals were subjected to 12 weeks of treatment.

### 2.6. Weight Changes

Body weight was assessed weekly on an electronic balance (Crystal 200, Gibertini, Italy). Weight change was calculated as the final weight minus the initial weight of the rats. After euthanasia, the liver was weighed.

### 2.7. Oxidative Stress Measurements

#### 2.7.1. Tissue Preparation

After 12 weeks of treatment, the animals were euthanized by decapitation, and the liver was quickly excised on a Petri dish, placed on ice. The liver was dissected and kept chilled until homogenization, which was performed using a ground glass-type Potter–Elvejhem homogenizer (Kimble Chase Life Science, Rockwood, TN, USA). Fresh tissue was homogenized in 1.5% KCl. The homogenates were centrifuged at 800× *g* for 10 min at 4 °C; the pellet was discarded, and the supernatants were kept at −70 °C until assays.

#### 2.7.2. Thiobarbituric Acid-Reactive Substances (TBARS) Measurement

Thiobarbituric acid-reactive substances (TBARS) were used to determine lipid peroxidation and were measured according to the method described by Ohkawa *et al.* (1979) [27]. Briefly, 50 μL of 8.1% sodium dodecyl sulfate (SDS), 375 μL of 20% acetic acid (pH 3.5) and 375 μL of 0.8% thiobarbituric acid (TBA) were added to 200 μL of homogenate, and the mixture was incubated in a boiling water bath for 60 min. After cooling, the mixture was centrifuged (1000× *g*, 10 min). The supernatant was removed, and the absorbance was read at 535 nm in a spectrophotometer (T80 UV/VIS Spectrometer, PG Instruments, Alma Parck, Lutterworth, UK). Commercially available malondialdehyde was used as a standard. Results were expressed as nmoL/mg protein.

### 2.8. Carbonyl Assay

A carbonyl assay was used to determine oxidative damage to proteins. Homogenates were incubated with 2,4 dinitrophenylhydrazine (DNPH, 10 mM) in 2.5 M HCl for 1 h at room temperature, in the dark. Samples were mixed every 15 min. Next, 20% (w/v) Trichloroacetic acid (TCA) was added to the tubes, which were then left in ice for 10 min and centrifuged for 5 min at 1000× *g*, to collect the protein precipitates. Another wash was performed with 10% TCA. The pellet was washed 3 times with ethanol:ethyl acetate (1:1) (v/v). The final precipitates were dissolved in 6 M guanidine hydrochloride, and the solutions were allowed to stand for 10 min at 37 °C and then read at 360 nm [28]. The results were expressed as nmoL/mg protein.

### 2.9. Sulfhydryl Assay

This assay is based on the reduction of 5,5′-dithio-bis(2-nitrobenzoic acid) (DTNB) by thiols, generating a yellow derivative (TNB), whose absorption is determined spectrophotometrically at 412 nm [29]. Briefly, 0.1 mM DTNB was added to 120 μL of the samples. This was followed by a 30-min incubation at room temperature in a dark room. Absorbance was measured at 412 nm. The sulfhydryl content is inversely correlated to oxidative damage to proteins. Results were reported as nmoL/mg protein.

### 2.10. Determination of Antioxidant Enzyme Activities

Superoxide dismutase (SOD) activity, expressed as Units of Superoxide dismutase (USOD/mg protein), was based on the decrease in the rate of autocatalytic adrenochrome formation at 480 nm [30]. Catalase (CAT) activity was determined by following the decrease in hydrogen peroxide (H_2_O_2_) absorbance at 240 nm and expressed as Units of Catalase (UCAT/mg protein) [31]. Glutathione peroxidase (GPx) activity was measured by following Nicotinamide adenine dinucleotide phosphate (NADPH) oxidation at 340 nm, as described by Flohe and Gunzler (1984) [32], and expressed as Units of Glutathione peroxidase (UGPx/mg protein).

### 2.11. 2′,7′-Dihydrodichlorofluorescein Oxidation Assay

Oxygen and nitrogen reactive species production was assessed according to LeBel *et al.* (1992) [33] by using reduced 2′,7′-diclorofluorescein diacetate (DCF-DA). Samples (30 μL) were incubated for 30 min at 37 °C in the dark with 30 μL of 20 mM sodium phosphate buffer, pH 7.4, with 140 mM KCl and 240 μL of 100 μM reduced 2′,7′-diacetate dichlorodihydrofluorescein (H_2_DCF-DA) in a 96-well plate. H_2_DCF-DA is cleaved by cellular esterases, and the 2′,7′-diclorofluorescin diacetate H_2_DCF formed is oxidized to DCF by reactive oxygen species (ROS) or reactive nitrogen species (RNS) present in the samples. DCF fluorescence intensity parallels the amount of reactive species formed. Fluorescence was measured using excitation and emission wavelengths of 480 and 535 nm, respectively. The calibration curve was prepared with standard DCF (0.25–10 μM), and the levels of reactive species were expressed as μmol DCF/mg protein.

### 2.12. Protein Determination

Protein concentrations were determined by the method of Lowry *et al.* (1951) [34] using bovine serum albumin as the standard.

### 2.13. Histopathological Analysis of the Liver Tissues

Part of the liver, especially the left lobe, was used for the histopathology analysis of the tissue cytoarchitecture and histochemistry as described by Normann *et al.* (2008) [35]. Briefly, the liver lobes were fixed in 10% buffered formalin at 4 °C for 24 h, dehydrated in an alcohol series, cleared in chloroform and embedded in paraffin. The histological sections of 7 μm were prepared using a Leica microtome (Leica RM 2155, Nussloch, Germany). For histological and cytoarchitechtonic evaluation, sections were stained with hematoxylin for 20 min and eosin for 2 min (HE).

### 2.14. Statistical Analysis

Data from the experiments were analyzed statistically by one-way analysis of variance (ANOVA) followed by the Tukey test. Values of *p* < 0.05 were considered significant. All analyses were carried out using the Statistical Package for Social Sciences (SPSS) software (version 17.0, International Business Machines Corporation, New York, NY, USA).

## 3. Results

### 3.1. Effect of Treatment with HFD and Grape Juices on Animal Body Composition

First, the body composition of the rats was assessed after 12 weeks of treatment with HFD, conventional grape juice and organic grape juice (Table 1). It was observed that the animals treated with HDF and conventional or organic grape juices had a lower body weight and that the animals supplemented with HFD and OGJ had lower weight gain. The liver weight was not altered by any of the treatments, while the ratio body weight/liver weight was lower in the rats treated with HFD and OGJ.

**Table 1:** Body and liver weight studies of animals treated with a high-fat diet (HFD), conventional grape juice (CGJ) and organic grape juice (OGJ).

Parameters	Control	HFD	HFD + CGJ	HFD + OGJ
Weight (g)	329.2 ± 16.04	315.4 ± 14.62	279.4 ± 4.95 *	274.3 ± 9.04 *
Weight gain (g)	274.4 ± 16.39	260.2 ± 14.44	230.6 ± 3.78	225.1 ± 9.1 *
Liver weight (g)	9.2 ± 0.64	9.0 ± 0.47	8.6 ± 0.2	8.7 ± 0.4
Body weight (g)/Liver weight (g)	36.0 ± 2.92	34.9 ± 3.54	32.5 ± 2.42	30.6 ± 2.50 *

Mean ± SD. One-way ANOVA, followed by Tukey test, * *p* < 0.05, different from the control; *n* = 10.

### 3.2. Effect of Treatment with HFD and Grape Juices on Oxidative Stress Parameters

**Figure 1:**
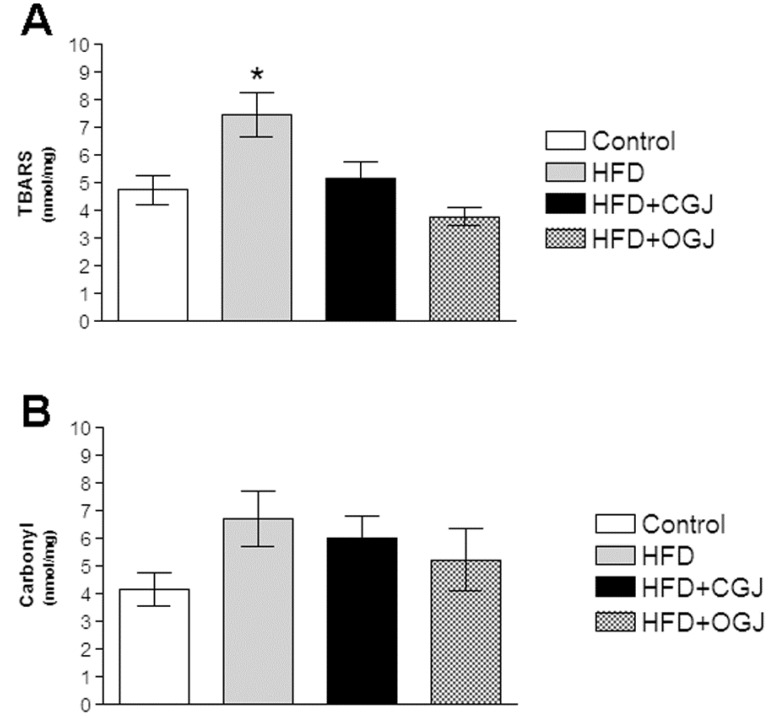
The effect of purple grape juices and high-fat diet on the formation of thiobarbituric acid-reactive substances (TBARS) (**A**) and carbonyl groups (**B**) in liver of rats. Values are the means ± SD for 8–10 samples in each group expressed as nmoL/mg. Statistically significant differences were determined by ANOVA followed by the Tukey test: * *p* < 0.05, different from the other groups. HFD, high-fat diet; CGJ, conventional grape juice; OGJ, organic grape juice.

Figure 1 demonstrates that the HFD enhanced lipid peroxidation without affecting protein oxidation (carbonyl) in the liver of rats. It was also found that conventional and organic grape juices both reduced TBARS levels (Figure 1A).

**Figure 2:**
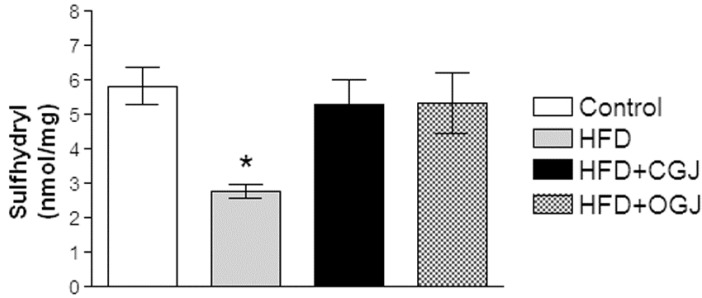
The effect of purple grape juices and high-fat diet on protein sulfhydryl groups in the liver of rats. Values are the means ± SD for 8–10 samples in each group expressed as nmoL/mg. Statistically significant differences were determined by ANOVA followed by the Tukey test: * *p* < 0.05, different from the other groups. HFD, high-fat diet; CGJ, conventional grape juice; OGJ, organic grape juice.

Next, the effect of HFD was observed on the non-enzymatic antioxidant defenses by measuring protein sulfhydryl groups. Figure 2 shows that sulfhydryl groups were decreased by this treatment and that both juices (conventional and organic) prevented this alteration in the liver of rats.

**Figure 3:**
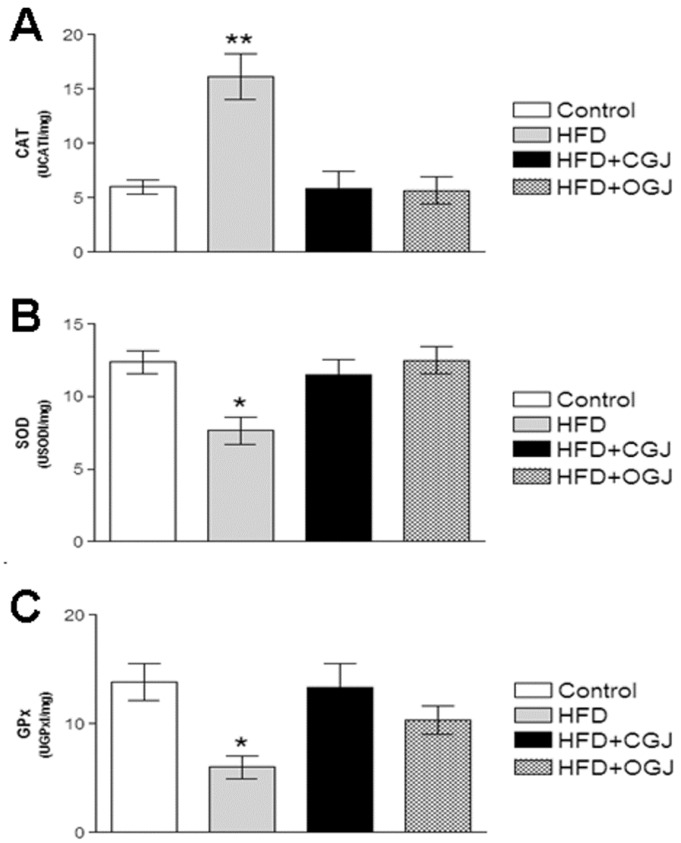
The effect of purple grape juices and high-fat diet on the activities of the antioxidant enzymes catalase (CAT) (**A**), superoxide dismutase (SOD) (**B**) and glutathione peroxidase (GPx) in the liver of rats. Values are means ± SD for 8–10 samples in each group. Statistically significant differences were determined by ANOVA followed by the Tukey test: * *p* < 0.05, ** *p* < 0.01 different from the other groups.

Moreover, the effect of HFD on the enzymatic antioxidant defenses was investigated by measuring CAT, SOD and GPx activities. Figure 3 shows that the CAT activity was increased by HFD, while the SOD and GPx activities were reduced in the liver of rats. Conventional and organic grape juices restored the activity of these enzymes.

**Figure 4:**
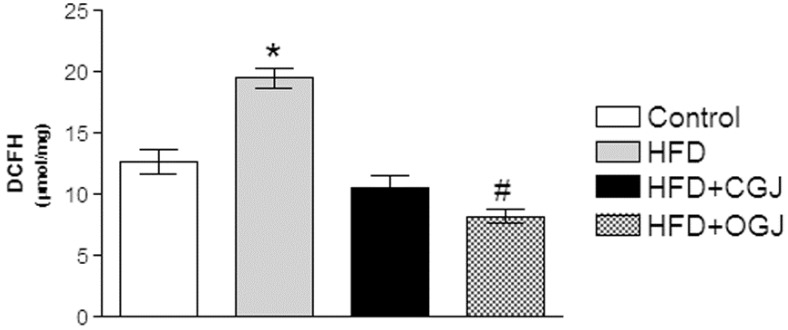
The effect of purple grape juices and high-fat diet on 2′,7′-dihydrodichlorofluorescein (DCFH) oxidation in the liver of rats. Values are the means ± SD for 8–10 samples in each group. Statistically significant differences were determined by ANOVA followed by the Tukey test: * *p* < 0.05, different from the other groups; ^#^ *p* < 0.05, different from the control.

HFD increased the reactive oxygen species, as indicated by the increase in the DCF levels, and this was prevented by treatment with either grape juice, where the organic one was more effective (Figure 4).

### 3.3. Effect of Treatment with HFD and Grape Juices on Cytoarchitecture and Histochemistry of Liver

**Figure 5:**
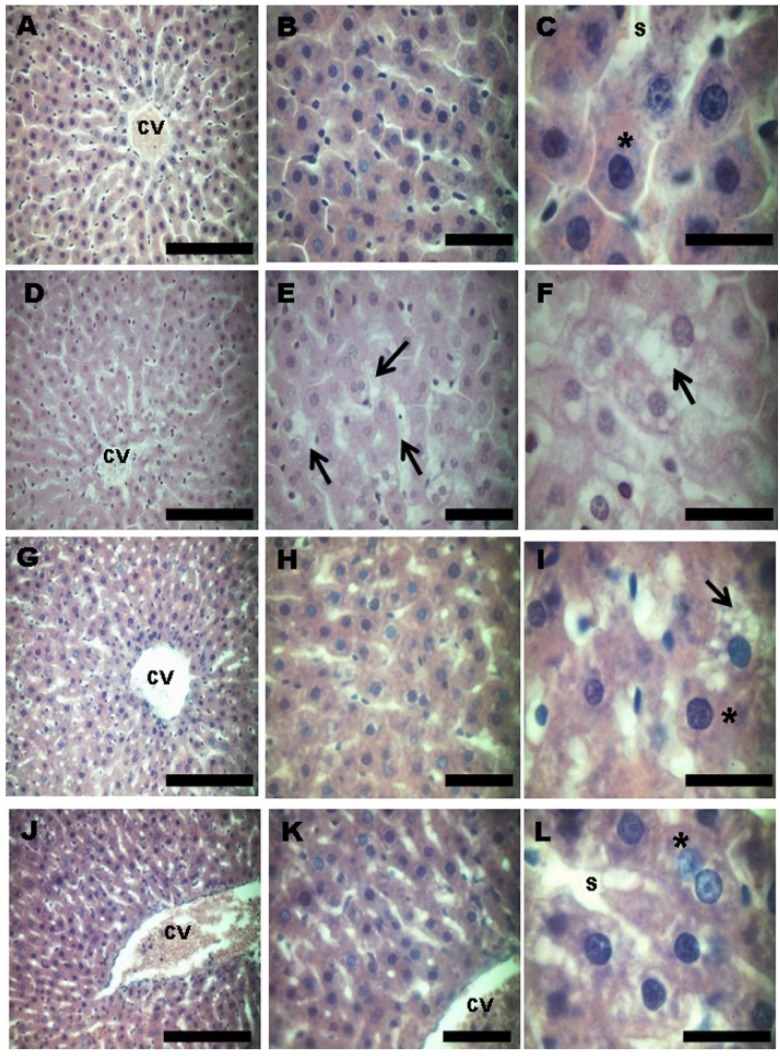
Photomicrographs (hematoxylin and eosin (HE)) showing the cytoarchitecture in the liver of rats treated with a high-fat diet and purple grape juices. (**A**–**C**) Control group, showing the characteristic architecture, with normal hepatocytes (asterisk). (**D**–**F**) The high-fat diet group, showing fatty changes of hepatocytes and steatosis with ballooning hepatocytes (arrow). (**G**–**I**) High-fat diet and conventional grape juice group, showing reduced fatty changes. Isolated ballooned hepatocytes (arrow) are seen differing from normal hepatocytes (asterisk), which represent the majority of liver cells. (**J**–**L**) High-fat diet and organic grape juice group, showing the same histological pattern as the control group. CV, central vein; S, sinusoid. Scale bar: A, D, G and J: 50 μm; B, E, H and K: 20 μm; C, F, I and L: 10 μm. The photomicrographs show the most representative slide of each group.

The morphological evaluation of the liver by HE staining showed that the control group had normal characteristics with normal hepatocytes (indicated by the asterisks) and no evidence of damage (Figure 5A–C). The HFD hepatocytes showed induced liver damage, with evidence of hepatocellular degeneration and steatosis (indicated by the arrows) (Figure 5D–F). The grape juice treatment markedly reduced the liver damage caused by HDF. In the conventional grape juice treatment, the majority of the cells were normal hepatocytes (asterisk), and only a few isolated cells with steatosis (arrows) were observed (Figure 5G–I). The liver cells of the animals treated with OGJ consisted of only a normal hepatocyte architecture with no evidence of damage or altered cells, showing the same histological pattern as the control group (Figure 5J–L).

## 4. Discussion

It is well described in the literature that HFD is one of the factors that induces obesity in humans and also in experimental animal models [1,36,37]. Besides inducing obesity, excessive intake of fat also supports the appearance of co-morbidities, such as hypertension, diabetes mellitus, hyperlipidemia and cardiovascular diseases [1,38]. As a consequence of increased body fat, weight and blood lipoproteins, oxidative stress may arise [39,40,41]. Some studies have shown that a healthy diet has balanced concentrations of proteins, carbohydrates, lipids, vitamins, minerals, fiber and water, which besides nurturing, can contribute to the prevention of co-morbidities [1,42]. Accordingly, the intake of fruits and vegetables also plays an important role in maintaining the physiological balance of the redox status. These foods have different antioxidant compounds capable of combating oxidative stress [21,43]. Therefore, the present study evaluated the antioxidant and hepatoprotective potential of the chronic use of CGJ and OGJ on oxidative stress in the liver of rats supplemented with HFD.

Grapes have been widely studied because of their antioxidant properties [9,21,44,45]. It is well described in the literature that grapes can afford protection against neurodegenerative and metabolic diseases [14,46,47,48,49]. Furthermore, some studies have also demonstrated the benefits of the compounds present in grapes in the liver of rats. The hydroalcoholic extract of black grapes was found to prevent lead-induced oxidative stress [50], while *Vitis labrusca* grape seed extract protected against oxidative damage to lipids and proteins induced by pentylenetetrazol [51]. *Vitis vinifera* ethanolic extract was found to have a significant protective effect by lowering the serum levels of alkaline phosphatase and total bilirubin [52], while resveratrol significantly decreased oxidative stress and hepatic inflammation (NF-κB and IL-1β ) in streptozotocin (STZ)-induced Type 1 diabetic rats [53].

The present study demonstrated that CGJ and OGJ were able to decrease animal weight, and OGJ was capable of reducing the weight gain and the body weight/liver weight/ratio in rats supplemented with HFD. The present data are in line with previous studies showing that mice fed HFD and grape phytochemicals had a significant decrease in body weight compared to HFD animals [54] and that the accumulation of abdominal white adipose tissue was markedly prevented in resveratrol diet-fed rats [55]. In this context, daily treatment with resveratrol lowered the weight in mice by increasing their aerobic capacity and inducing genes for oxidative phosphorylation and mitochondrial biogenesis, which protected mice against diet-induced-obesity and insulin resistance [56]. This is in line with the present study, where OGJ was more effective in decreasing weight gain and that this effect could be due to the higher resveratrol and polyphenol content of this grape juice.

Here, it was observed that HDF induced oxidative stress in the liver of rats by increasing lipid peroxidation (TBARS), reducing non-enzymatic antioxidant defenses (sulfhydryl content) and compromising enzymatic antioxidant defenses by increasing CAT, reducing SOD and GPx activity and enhancing DCHF levels. These data corroborated the findings of Du *et al.* (2012) [57], who reported that HFD significantly increased the ROS generation and the expression of NADPH oxidase and uncoupling proteins in rats, suggesting mitochondrial damage in response to excessive fat intake. Moreover, Fachinetto *et al.* (2005) [58] and Ribeiro *et al.* (2009) [59] described that HFD supplement was associated with an increase in TBARS levels in the brain of rats fed HFD, and Muthulakshmi and Saravan (2013) [60] described a decrease in enzymatic and non-enzymatic antioxidant defenses along with a significant increase in lipid peroxidation markers in the liver of HFD-fed mice.

It was also demonstrated that CGJ and OGJ were able to prevent the oxidative stress caused by HFD in the liver of rats. This is in line with previous studies demonstrating that purple grape juice (organic and conventional) was able to prevent the increase in TBARS and carbonyl groups, antioxidant enzymes SOD and CAT and the reduced sulfhydryl content induced by pentylenetetrazol in brain, liver and serum of rats [20,61]. Dani *et al.* (2008) [62] also showed that purple grape juice prevented the damage caused by carbon tetrachloride (CCl_4_) in tissues of rats. These studies attributed this prevention to the rich polyphenol content of grapes.

The present study showed that HFD induced hepatocellular degeneration and steatosis and CGJ and OGJ reduced the liver damage, where OGJ was more effective in this protection. It was previously found that *Vitis vinifera* extract was able to significantly reduce CCl_4_-associated ballooning degeneration and apoptotic cell counts in the liver of rats [63]. Moreover, in earlier studies, naringenin, a bioflavonoid present in grapes, and vitamins C and E significantly improved the altered biochemical and histopathological changes in the liver of cadmium-intoxicated rats [64]. Oral intake of grape seeds (*Vitis vinifera*) attenuated histopathological changes caused by tamoxifen in the liver of rats [65], and resveratrol reduced the hepatic stellate cell activation and hepatic fibrosis induced by dimethylnitrosamine in rats [66].

## 5. Conclusions

Taken together, HFD induced lipid peroxidation, compromised the non-enzymatic and enzymatic antioxidant defenses, increased the levels of reactive species and induced hepatocellular degeneration and steatosis in the liver of rats. Considering that grape juice, a complex mixture rich in polyphenol content and vitamins, was able to prevent these alterations, we speculate that regular intake of grape products could be considered an adjuvant in the therapy of patients with metabolic and hepatic diseases
